# 5mC-hydroxylase activity is influenced by the PARylation of TET1 enzyme

**DOI:** 10.18632/oncotarget.4476

**Published:** 2015-06-15

**Authors:** Fabio Ciccarone, Elisabetta Valentini, Michele Zampieri, Paola Caiafa

**Affiliations:** ^1^ Department of Cellular Biotechnologies and Hematology, “Sapienza” University of Rome and Pasteur Institute-Fondazione Cenci Bolognetti, Rome, Italy

**Keywords:** PARylation, TET1, 5hmC

## Abstract

5-hydroxymethylcytosine is a new epigenetic modification deriving from the oxidation of 5-methylcytosine by the TET hydroxylase enzymes. DNA hydroxymethylation drives DNA demethylation events and is involved in the control of gene expression. Deregulation of TET enzymes causes developmental defects and is associated with pathological conditions such as cancer. Little information thus far is available on the regulation of TET activity by post-translational modifications. Here we show that TET1 protein is able to interact with PARP-1/ARTD1 enzyme and is target of both noncovalent and covalent PARylation. In particular, we have demonstrated that the noncovalent binding of ADP-ribose polymers with TET1 catalytic domain decreases TET1 hydroxylase activity while the covalent PARylation stimulates TET1 enzyme. In addition, TET1 activates PARP-1/ARTD1 independently of DNA breaks. Collectively, our results highlight a complex interplay between PARylation and TET1 which may be helpful in coordinating the multiple biological roles played by 5-hydroxymethylcytosine and TET proteins.

## INTRODUCTION

Poly(ADP-ribosyl)ation (PARylation) is a post-translational modification catalyzed by enzymes of the poly(ADP-ribose) polymerase (PARP) family, whose founding member is PARP-1 also known as ADP-ribosyltransferase diphtheria toxin-like 1 (ARTD1) [1]. PARPs use NAD^+^ as substrate producing negatively charged polymers of ADP-ribose (PARs) which are then hydrolyzed by the poly(ADP-ribose) glycohydrolase (PARG) [2-4]. DNA damage highly stimulates the activity of PARP-1/ARTD1 [5, 6], which functions as DNA break sensor PARylating DNA damage response effectors [7-10]. However, secondary DNA structures and allosteric trans-activating factors can also trigger PARylation [6, 11, 12] supporting other housekeeping functions of PARP-1/ARTD1 such as transcriptional regulation.

Besides the covalent PARylation of target proteins, PARP-1/ARTD1 itself and other PARP family members typically undergo automodification reaction [3, 6]. Moreover, target proteins bringing specific PAR-interacting motifs are able to accommodate PARs noncovalently [4, 13]. All these features of PARylation permit PARP-1/ARTD1 to modulate protein-protein interactions, subcellular localization or enzymatic activities [14-19]. Therefore, PARylation participates in a variety of cellular processes including DNA damage response, transcription and apoptosis [3, 16]. PARP-1/ARTD1 regulates gene expression acting on histones [20, 21], transcriptional factors (e.g. CTCF, SOX2) [22, 23] or proteins involved in chromatin dynamics (e.g. ISWI, HP1) [24, 25]. Furthermore, PARP-1/ARTD1 is able to directly influence epigenetic events [26] also through the modification of enzymes involved in histone post-translational modifications (e.g. KDM5B, KDM4D) [17, 27] or in the regulation of DNA methylation patterns (e.g. DNMT1, UHRF1) [19, 28].

As concerns DNA methylation, apart from inhibiting DNMT1 enzymatic activity by noncovalent PARylation [19], PARylated PARP-1/ARTD1 positively controls *DNMT1* expression [29]. Recently, we have demonstrated that PARP activity is involved in the transcriptional regulation of the *Ten-eleven-translocation 1* (*TET1*) gene [30, 31] codifying for an enzyme that introduces the 5-hydroxymethylcytosine (5hmC), a new epigenetic modification present on DNA.

5hmC derives from the oxidation of 5-methylcytosine (5mC) through the action of the 2-oxoglutarate/Fe(II)-dependent DNA dioxygenases TET1, TET2 and TET3 [32]. 5hmC can function as an intermediate of both passive and active DNA demethylation processes [33, 34]. The action of 5hmC in passive DNA demethylation seems to depend on the reduced binding affinity of the maintenance methylase DNMT1 for 5hmC [35]. As concerns active DNA demethylation, the presence of 5hmC is a prerequisite for sequential oxidation reactions, and even they are catalyzed by TET enzymes leading to formation of 5-formylcytosine (5fC) and 5-carboxylcytosine (5caC) [36]. Thymine DNA glycosylase (TDG)-mediated base excision repair (BER) has a pivotal role in the removal of 5fC and 5caC and re-introduction of unmethylated cytosine [37]. However, 5hmC is now considered as the sixth base of DNA introducing an additional epigenetic code onto genome [32, 38]. In mammals, the levels of 5hmC are different in tissues and cell types. Brain and embryonic stem cells show the highest abundance of DNA hydroxymethylation [39, 40]. 5hmC is depleted in stable heterochromatic regions, while it is frequently associated with promoter-proximal regions, enhancers or transcription factor binding sites. Moreover, 5hmC is particularly enriched in CpG islands (CGIs) with low to medium GC-content but it is depleted in strong CGI which are generally completely unmethylated [41-44]. Genomic distribution of 5hmC mainly concentrated in proximity of coding sequences or of distal regulative elements supports the involvement of 5hmC in transcriptional regulation. Accordingly, a number of proteins display binding preference towards 5hmC thus functioning as readers of this modification and interpreters of the 5hmC epigenetic code. Notably, specific binders of 5fC or 5caC have also been identified [45, 46] and recent evidence has suggested the involvement of these additional DNA modifications in the regulation of transcription influencing RNA polymerase II activity as well as DNA methylation dynamics on active promoters [47-49]. Besides a role of PARylation in preserving a permissive chromatin state on *TET1* gene promoter [31, 50], an involvement of PARs has also been demonstrated for the recruitment of TET1 protein onto specific *loci* during adipocyte differentiation [51]. Considering the multiple ways of action of PARylation in the regulation of protein functions [6, 16], we decided to investigate further the interplay between TET1 and PARP-1/ARTD1. All in all, our results highlighted that TET1 is a target of both covalent and noncovalent PARylation with consequences on TET enzymatic activity and that TET1 is in itself able to stimulate PARP-1/ARTD1 activation.

## RESULTS

### PARP inhibition affects TET1-mediated 5hmC formation

**Figure 1:**
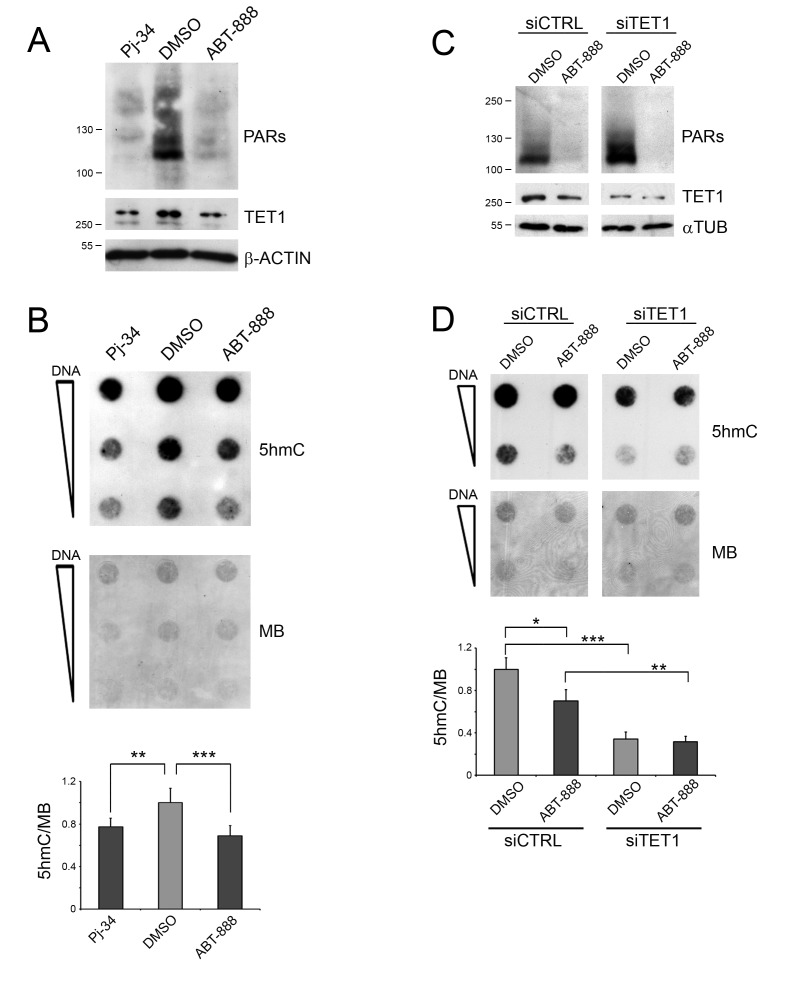
Inhibition of PARP activity affects TET1-dependent 5hmC formation **A.** Western blot analysis showing the effect of PARP inhibition on HEK293T cells treated with Pj-34 and ABT-888 for 72 hrs. **B.** 5hmC dot-blot analysis after inhibition of PARylation for 72 hrs and relative quantification. Results are shown as means ± S.E.M. (*n* = 5). **C.** Western blot analysis showing the silencing of TET1 and the levels of PARs after ABT-888 treatment. **D.** 5hmC dot-blot analysis and relative quantification after inhibition of PARylation for 72 hrs in control (siCTRL) and TET1-silenced (siTET1) cells. Results are shown as means ± S.E.M. (*n* = 4). Quantification of 5hmC levels was performed by densitometric analysis using methylene blue (MB) staining as DNA loading control. *P*-values were determined by ANOVA with post hoc Tukey's test (*P < 0.05; **P < 0.01; ***P < 0.001).

HEK293T cells were treated with two competitive inhibitors of PARP activity, Pj-34 and ABT-888. Both PARP inhibitors provoked the disappearance of PAR levels which was associated with a reduction of TET1 protein (Figure 1A). The transcriptional analysis of the main genes codifying for PARP machinery members (i.e. PARP-1, PARP-2, PARP-3 and PARG) showed no differences after PAR depletion (Supplementary Figure S1). Dot-blot and ELISA-based 5hmC quantification analyses evidenced that the inhibition of PARP activity caused a moderate reduction of the global content of 5hmC with respect to control cells (Figure 1B and Supplementary Figure S2A). The silencing of TET1 (Figure 1C) was performed to analyse the involvement of TET1 activity in the formation of 5hmC in HEK293T and its contribution to the effects mediated by PARP inhibition. 5hmC dot-blot analysis showed that silencing of TET1 markedly decreases the formation of 5hmC in HEK293T with respect to CTRL-silenced cells. Notably, the effect of PARP inhibition on 5hmC formation was no longer evident after the silencing of TET1 indicating that TET1 protein has a major role in this phenomenon in HEK293T cells (Figure 1D).

### The action of PARylation on TET1 enzyme is not limited to protein recruitment

**Figure 2:**
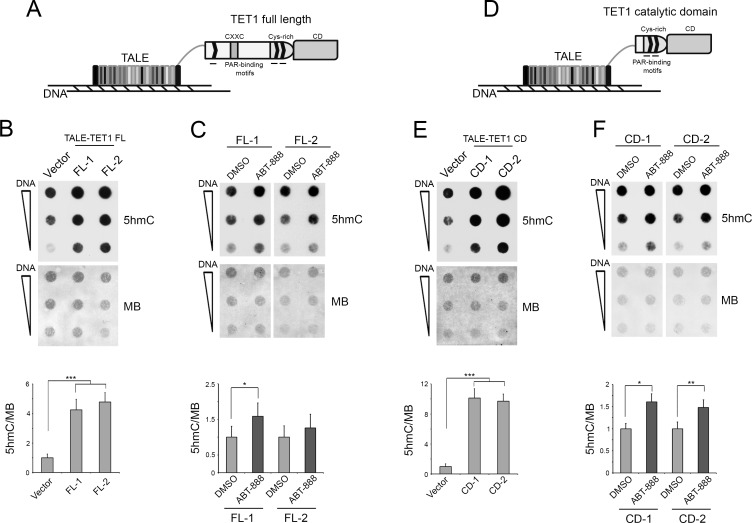
The levels of 5hmC, deriving from TALE-TET1 protein overexpression, increase after PARP inhibition **A.** Schematic illustrating the TALE fused to TET1 full-length protein (TET1 FL) containing the CXXC-type zinc-binding domain (CXXC), the cysteine-rich region (Cys-rich), the catalytic domain (CD) and the PAR-binding motifs. **B.** Dot-blot analysis of 5hmC after overexpression of two different TALE-TET1 FL (FL-1 and FL-2) proteins for 72 hrs. Results are shown as means ± S.E.M. (*n* = 3) **C.** Dot-blot analysis of 5hmC after overexpression of FL-1 and FL-2 and inhibition of PARP activity. Results are shown as means ± S.E.M. (*n* = 3). **D.** Schematic illustrating the TALE fused to the catalytic domain of TET1 protein (TET1 CD) containing the cysteine-rich region (Cys-rich), the catalytic domain (CD) and the PAR-binding motifs. **E.** Dot-blot analysis of 5hmC after overexpression of two different TALE-TET1 CD (CD-1 and CD-2) proteins for 48 hrs. Results are shown as means ± S.E.M. (*n* = 3). **F.** Dot-blot analysis of 5hmC after overexpression of CD-1 and CD-2 and inhibition of PARP activity. Results are shown as means ± S.E.M. (*n* = 3). Quantification of 5hmC levels was performed by densitometric analysis using methylene blue (MB) staining as DNA loading control. P-values were determined by ANOVA with post hoc Tukey's test or paired Student *t*-test (*P < 0.05; **P < 0.01; ***P < 0.001).

Engineered transcription activator-like effector (TALE) is customizable DNA-binding domain designed to target specific sites on genome [52]. We decided to use TALEs fused to TET1 protein [53] to obtain a recruitment of TET1 onto DNA independently of PARylation (Figure 2A). In fact, the noncovalent PARylation of murine TET1 has been described as being involved in the recruitment of this protein on specific *loci* during adipocyte differentiation [51]. Being TALE constructs fused to the human TET1 protein, we confirmed the conservation of putative PAR-binding motifs in it. Moreover, we identified an additional site for noncovalent PARylation in an aminoacid sequence of the human TET1 catalytic domain absent from the murine TET1 protein (Supplementary Figure S3).

Blast analysis of two sequences recognized by two different TALE-TET1 full-length proteins (FL-1 and FL-2) showed that similar DNA regions are randomly distributed on genome (data not shown). According to this, a global increase of 5hmC levels was effectively evidenced after overexpression of FL-1 or FL-2 TALE-TET1 proteins (Figure 2B and Supplementary Figure S4A). Notably, treatment with the PARP inhibitor ABT-888 of HEK293T cells overexpressing TALE-TET1 FL induced new increase of 5hmC levels with respect to untreated cells (Figure 2C and Supplementary Figure S2B). These results highlight the existence of additional roles played by PARylation on 5hmC formation, which are independent of TET1 recruitment on genome. To investigate a possible regulation of TET activity mediated by PARylation, two different TALE proteins fused only to TET1 catalytic domain (CD-1 and CD-2) were overexpressed (Figure 2D and Supplementary Figure S4B). Also TALE-TET1 CD overexpression caused a global increase of 5hmC (Figure 2E), which even in this case was more evident after the inhibition of PARP activity (Figure 2F and Supplementary Figure S2C).

### TET1 is a protein partner of PARP-1/ARTD1

**Figure 3:**
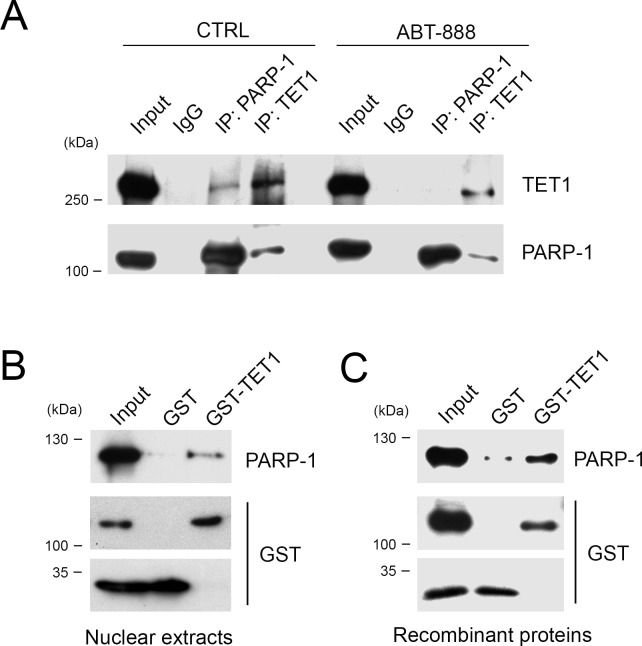
TET1 interacts with PARP-1 protein also in absence of PARs **A.** Co-Immunoprecipitation experiments performed with anti TET1 or anti PARP-1 antibodies on nuclear lysates of HEK293T treated or not with ABT-888 for 6 hrs. Input lysate was 10% of total. **B.** GST pull-down performed by using GST-TET1 and GST as control in presence of HEK293T nuclear lysates or **C.** recombinant PARP-1 protein.

A possible direct influence of PARylation on TET1 activity would imply an interaction between PARP-1 and TET1 proteins. Therefore, co-immunoprecipitation experiments were performed by using anti TET1 or anti PARP-1 antibodies demonstrating the binding between these proteins. Notably, after treatment with the PARP inhibitor ABT-888, the binding between TET1 and PARP-1 was partially affected suggesting that it is stabilized by PARs (Figure 3A).

Further demonstration of the interaction between TET1 and PARP-1 was obtained through GST pull-down experiments by using a GST-tag fused to the catalytic domain of TET1 (GST-TET1) presenting the PAR-binding motif typical of the human protein. Incubation of GST-TET1 with HEK293T nuclear extracts evidenced that endogenous PARP-1 is able to bind the C-terminal catalytic domain of TET1 (Figure 3B). GST pull-down was also performed in presence of recombinant PARP-1 indicating that the binding to TET1 is actually direct (Figure 3C).

### TET1 protein binds PARs noncovalently

**Figure 4:**
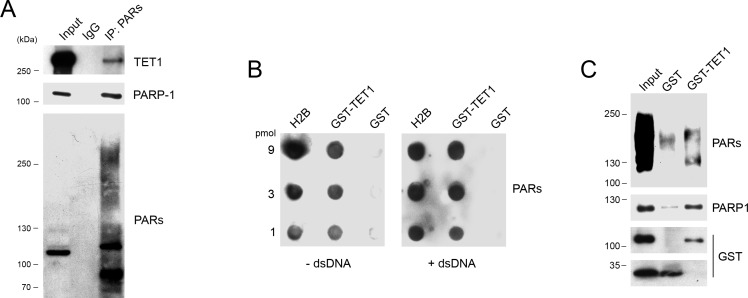
TET1 interacts noncovalently with PARs **A.** Immunoprecipitation experiments performed with anti PAR antibody on HEK293T nuclear lysates. Input lysate was 10% of total. **B.** PAR blot assay of GST-TET1, GST alone (negative control) and histone H2B (positive control) incubated with free PARs with/without dsDNA as competitor. **C.** GST pull-down performed by using GST-TET1 and GST as control in presence of automodified PARP-1 protein.

Immunoprecipitation experiments were performed with anti PAR antibodies confirming that endogenous TET1 is a target of PARylation (Figure 4A). *In vitro* PAR-binding assays with either free PARs or PARP-1 attached PARs were performed to demonstrate that TET1 catalytic domain is able to bind PARs noncovalently. PAR blot assay was carried out spotting 3-fold serial dilution of GST-TET1, histone H2B as positive control and GST-tag as negative control. Incubation with free PARs followed by anti PAR immunoblotting demonstrated the capacity of TET1 for binding PARs noncovalently. Notably, the interaction between PARs and TET1 catalytic domain persists even in presence of an excess of competitor double-stranded DNA (dsDNA) used to rule out non-specific interactions due to the highly negatively charged nature of PARs (Figure 4B). GST pull-down experiments performed by using *in vitro* automodified PARP-1 demonstrated that TET1 is able to bind PARs even when they are present on PARP-1 protein (Figure 4C).

### Inhibitory effect of noncovalent PARylation on TET1 activity

**Figure 5:**
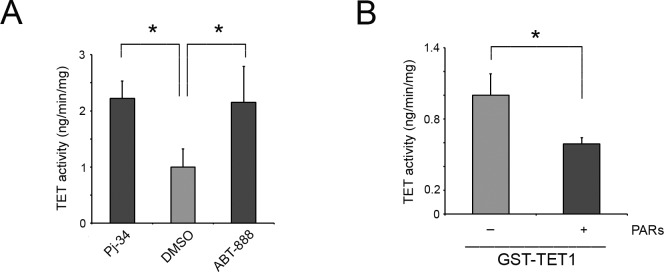
TET1 activity is negatively influenced by noncovalent PARylation **A.** 5mC-hydroxylase TET activity measured in nuclear extracts of HEK293T treated or not with PARP inhibitors for 6 hrs. Results are shown as means ± S.E.M. (*n* = 3). **B.** 5mC-hydroxylase TET activity of recombinant GST-TET1 enzyme measured in presence/absence of PARs. Results are shown as means ± S.E.M. (*n* = 5). P-values were determined by ANOVA with post hoc Tukey's test or paired Student *t*-test (*P < 0.05).

To verify whether PARylation is effectively able to influence TET1 hydroxylase activity, HEK293T cells were treated with the PARP inhibitors Pj-34 and ABT-888 (Supplementary Figure S5) and TET activity was measured *in vitro*. An increase of total TET activity was evidenced in absence of PARylation thus suggesting an inhibitory role of PARs on TET enzymes (Figure 5A).

To test the effect of noncovalent PARylation on TET1 enzymatic activity, GST-TET1 was incubated with PARs synthetized *in vitro* and purified. Notably, a reduction of TET1 hydroxylase activity was observed in presence of PARs suggesting that the noncovalent PARylation of TET1 catalytic domain is able to inhibit TET1 activity (Figure 5B).

### TET1 is covalently PARylated

**Figure 6:**
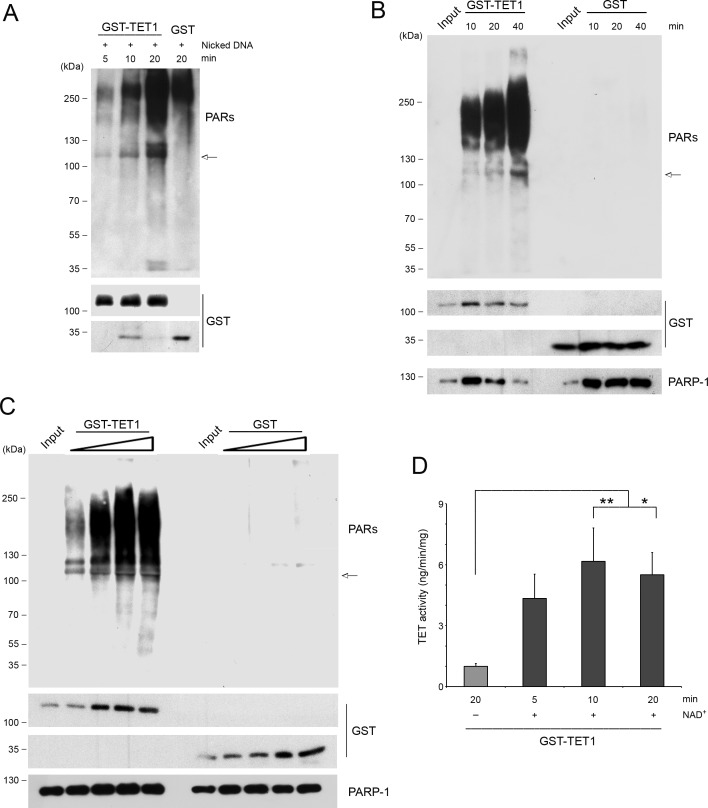
TET1 is covalently PARylated and is able to activate PARP-1 **A.** *In vitro* PARylation assay performed incubating PARP-1 with GST-TET1 for different times or with GST alone (negative control), in presence of nicked DNA added to stimulate PARP activity. **B.** *In vitro* PARylation assay in absence of nicked DNA performed by using PARP-1 with GST-TET1 or with GST alone (negative control) for different times or **C.** at different concentrations. **D.** 5mC-hydroxylase TET activity of GST-TET1 covalently modified by PARP-1. Results are shown as means ± S.E.M. (*n* = 4). P-values were determined by ANOVA with post hoc Tukey's test (*P < 0.05, **P < 0.01). Arrows indicate the bands revealed by anti PAR antibodies and corresponding to GST-TET1 molecular weight.

Besides noncovalent PARylation, proteins can also be covalently modified by PARs. To test whether TET1 can also be a target of covalent PARylation, an *in vitro* PARP assay was performed incubating GST-TET1 with recombinant PARP-1, NAD^+^ and nicked DNA to induce PARP activation. Western blot analysis with anti PAR antibodies evidenced time-dependent smears corresponding to PARP-1 activation. Moreover, the anti PAR antibody also revealed bands coincident with GST-TET1 molecular weight (arrow) indicating that TET1 is covalently PARylated *in vitro*. Notably, GST-TET1 sample incubated for 20 min with PARP-1 showed a more intense high molecular weight smear obtained with anti PAR antibody than the corresponding GST control sample suggesting that TET1 protein may have in itself the capacity for stimulating PARP activity (Figure 6A). Based on this, additional experiments were performed incubating GST-TET1 or GST alone with PARP-1 but in absence of activating nicked DNA. The incubation of PARP-1 with GST-TET1 for different times (Figure 6B) or with different concentrations of GST-TET1 (Figure 6C) showed a time- and dose-dependent increase of anti PAR signal, respectively. These results allowed the demonstration that TET1 is effectively able to activate PARP-1 independently of DNA damage. Notably, the presence of bands coincident with GST-TET1 molecular weight (arrow) detectable with anti PAR antibodies indicated that following the stimulation of PARylation by TET1, PARP-1 is in turn able to covalently modify TET1 protein (Figure 6B and 6C). The possibility that the covalent modification of TET1 may modulate the activity of TET1 protein was tested purifying PARylated GST-TET1 and measuring its hydroxylase activity *in vitro*. This assay suggested that the covalent PARylation of TET1 stimulates TET enzymatic activity (Figure 6D).

## DISCUSSION

The complexity of events involving 5hmC and its derivatives implies a fine-tuned regulation of TET protein activities to coordinate their actions [54]. Post-translational modifications are typically known to expand the range of functions of protein influencing subcellular localization, protein-protein or protein-nucleic acid interactions as well as enzymatic activities. The most studied modification of TETs is certainly the O-linked glycosylation catalysed by the O-linked N-acetylglucosamine (O-GlcNAc) transferase (OGT), which is able to modify all TET proteins but with different functional outcomes [55]. For example, while TET1 glycosylation seems to enhance protein stability [56], TET3 glycosylation regulates the subcellular localization [57]. Glycosylation of TETs also affects their phosphorylation which has recently been identified on TET proteins but whose function is still unknown [58]. In parallel, TET proteins are able to influence OGT activity with consequences on histone modifications and transcription [59].

An interesting link has also been observed between TET enzymes and PARylation [31, 51, 60]. TET1 is able to bind PARs noncovalently as it brings a PAR-binding motif within the N-terminal domain and another two motifs adjacent to the catalytic domain. The noncovalent PARylation of TET1 was demonstrated to be involved in the recruitment of TET1 onto specific *loci* by a PARylated protein complex comprising also PPARγ, which is the key regulatory factor in adipogenesis [51]. In addition, we have recently demonstrated a transcriptional control of *TET1* gene mediated by PARylation [31], result also confirmed by another group [50]. The well-known ability of PARP-1/ARTD1 to regulate protein functions by several modes [6, 16] prompted us to investigate further the interplay between PARylation and 5hmC formation focusing on TET1 enzymatic activity.

The use of TALE-TET1 fusion proteins (TET1 full-length or catalytic domain) highlighted new roles played by PARylation on TET1 function independently of protein recruitment which now is mediated by TALEs. Their overexpression induced an increase of 5hmC levels which was even more evident in presence of PARP inhibitors. The increase of 5hmC formation cannot be justified by the involvement of PARylation in the control of TET1 expression or recruitment onto genome. Rather, this finding suggests that PARs could suppress TET1 enzymatic activity as also indicated by the use of TALE constructs fused to the sole catalytic domain of TET1. Notably, TALE-TET1CD possesses PAR-binding motifs which interacting noncovalently with PARs may inhibit TET hydroxylase activity. The hypothesis of an inhibitory effect of PARs on TET enzymatic activity was validated measuring the total TET activity in cells after inhibition of PARylation. In addition, after the confirmation of a strong noncovalent interaction between PARs and TET1, the incubation of recombinant TET1 catalytic domain with PARs synthesised *in vitro* indicated that noncovalent PARylation of TET1 is effectively involved in the enzymatic repression. Further co-IP and pull-down experiments demonstrated an interaction between TET1 and PARP-1/ARTD1 which seems to be strengthened by PARs even though the proteins can also interact independently.

The identification of *in vitro* inhibitory action of noncovalent PARylation on TET1 enzyme is apparently in disagreement with the reduction of 5hmC levels observed after treatment of HEK293T cells with PARP inhibitors. Such *in vivo* effect of PAR depletion on genomic DNA is most likely to be the net result which also depends on the decrease of TET1 expression as well as on the failure of TET1 recruitment onto DNA mediated by PARs.

PARP-1/ARTD1 can modify target proteins covalently on glutamate, aspartate or lysine residues [61] and some proteins can undergo both covalent and noncovalent PARylation [10, 15, 22, 62]. *In vitro* experiments showed that TET1 can indeed be covalently modified by PARP-1/ARTD1 and this modification seems to have a different outcome on TET1 activity resulting in a stimulation of the enzyme. Notably, TET1 and PARP-1/ARTD1 are also connected by the ability of TET1 to trigger PARylation *in vitro* in absence of activating nicked DNA. In this context, the covalent modification of TET1 has also been evidenced.

Collectively, these results enlarge the complexity of the cross-talk existing between TET1 and PARP-1/ARTD1. In fact, apart from the involvement of PARylation in the transcriptional regulation of *TET1* gene [31, 50] and recruitment of TET1 protein onto specific *loci* [51], our findings highlight for the first time a direct control of PARs over TET1 hydroxylase activity. In particular, a bimodal influence of noncovalent and covalent PARylation emerges on the regulation of TET1 enzymatic activity. These multiple connections between PARP-1/ARTD1 and TET1 are not surprising considering the different ways adopted by PARylation in the regulation of protein functions [6, 16] and above all the numerous roles played by TET1 in the regulation of epigenetic dynamics [54].

Accordingly, 5hmC can be an intermediate of both active and passive DNA demethylation [33, 34]. In particular, TET enzyme activity is not limited to the formation of 5hmC but it is also responsible for the transformation of 5hmC in its derivatives, 5fC and 5caC, during the active DNA demethylation process [36, 37]. Besides the well-known involvement of PARP-1/ARTD1 in the BER pathway [3] which leads the reintroduction of unmethylated cytosines, a contribution of PARylation in the control of the sequential transformations of 5mC into 5hmC, 5fC or 5caC mediated by TETs can be suggested. Moreover, during active DNA demethylation the PARylation of TET1 itself could favour the assemblage of those DNA repair effectors known to be recruited on damaged DNA by PAR noncovalent interaction [8, 9, 13].

**Figure 7:**
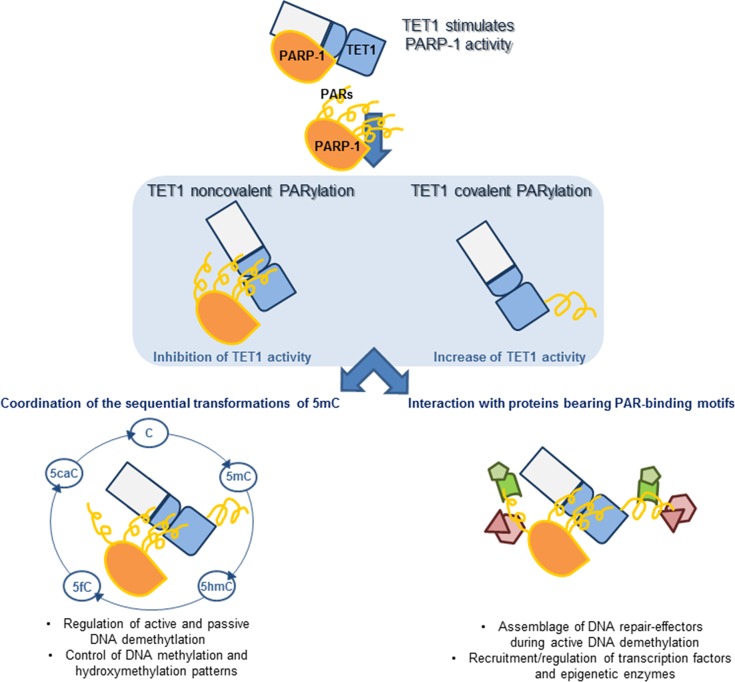
Model summarizing the possible outcomes of covalent and noncovalent PARylation of TET1 on its biological functions TET1 can stimulate the activity of PARP-1 independently of DNA damage. In turn, TET1 can be PARylated by PARP-1 both noncovalently and covalently with consequences on TET1 activity. PARylation of TET1 may regulate the hydroxylase activity during the different steps of the DNA demethylation processes. Apart from the regulation of TET activity, PARylation of TET1 may act in the assemblage of complexes containing PAR-interacting proteins involved in the regulation of DNA demethylation and transcription.

It is noteworthy that TET1 is involved in transcriptional regulation even independently of its hydroxylase activity. This is also supported by the evidence that TET1, by its CXXC domain, preferentially binds to unmehylated CGIs where the hydroxylase activity is not required missing the 5mC substrate [63-65]. In this context, PARylated TET1 could limit the access of DNMT1 onto DNA preventing DNA methylation [19, 29, 64] and it can favour the binding of transcription factors that bind PARs noncovalently (Figure 7).

In conclusion, in this work we have identified for the first time a mechanism involved in the direct regulation of TET enzymatic activity mediated by PARylation. A deeper characterization of the interplay between TET1 and PARP-1/ARTD1 also in relation with other post-translational modifications would highlight new mechanisms driving TET1 functions. This is relevant if considering that TET enzymes and 5hmC are not only involved in development and cell differentiation [34] but also in several pathological conditions including cancer [66-68] and neurodegenerative disorders [69-71].

## MATERIALS AND METHODS

### Cell culture, treatment and transfection

HEK293T cells were grown in high glucose DMEM (Sigma-Aldrich) containing 10% FBS (Sigma-Aldrich), 2 mM L-glutamine (Sigma-Aldrich) and 50 U/ml Penicillin–Streptomycin (Sigma-Aldrich). Treatments of cells were performed replacing medium every 24 hrs with the PARP inhibitors PJ-34 (Sigma-Aldrich, final concentration 1 μM), and ABT-888 (Enzo Life Sciences, final concentration 1 μM). Transfection of HEK293T cells was performed by using Lipofectamine 2000 reagent (Life Technologies) adopting the manufacturer's protocol. TET1 silencing was obtained by using DsiRNA Duplex for TET1 gene (IDT Integrated DNA technologies, final concentration 10nM). TALE-TET1 overexpression plasmids namely JA740 (Addgene plasmid # 49939), SL357 (Addgene plasmid # 49936), MLM3713 (Addgene plasmid # 49946), MLM3727 (Addgene plasmid # 49961) were a gift from Keith Joung. In the manuscript, JA740, SL357, MLM3713, MLM3727 plasmids correspond to FL-1, FL-2, CD-1, CD-2.

### Antibodies

The following monoclonal antibodies were used: PARP-1 (clone C2-10; Enzo Life Sciences), PAR (clone 10HA; Trevigen), TET1 (Genetex), Myc-tag (9E10 clone, hybridoma-conditioned medium), GST (Thermo), αTUB (Sigma-Aldrich), FLAG (Thermo Scientific Pierce Antibodies). The following polyclonal antibodies were used: PARP-1 (Enzo Life Sciences), PAR (10H, kind gift of A. Burkle), 5hmC (Active motif), Lamin B1 (Abcam).

### Western blot analysis

Total cell lysates were prepared in RIPA buffer (50 mM Tris-HCl at pH 7.4, 150 mM NaCl, 1% NP-40, 0.5% sodium deoxycholate, 0.1% SDS and 1 mM EDTA) and normalized for protein concentration. Nuclei were obtained after incubation of cells for 15 min in ice with isolation buffer (10 mM Tris-HCl at pH 7.8, 4 mM MgCl_2_, 1 mM EDTA, 0.5 mM DTT, 1% Triton X-100, 0.25 M Sucrose); pelleted nuclei were washed with isolation buffer without Triton X-100 and centrifuged. Each buffer was supplemented with protease inhibitor cocktail (Complete EDTA-free, Roche Applied Science). Protein extracts were resolved by SDS-PAGE in 6% acrylamide/bis-acrylamide gels, transferred onto Hybond-ECL nitrocellulose membranes (Amersham Biosciences) and probed with the indicated antibodies.

### Dot blot assay

DNA was extracted with DNeasy Blood & Tissue Kit (QIAGEN), denatured in 0.4 M NaOH, 10mM EDTA at 95°C for 10 min and then neutralized by adding an equal volume of cold 4 M ammonium acetate (pH 7.0). 2-fold dilutions of denatured DNA samples were spotted on nitrocellulose membrane Hybond-N^+^ (Amersham Biosciences) in an assembled Bio-Dot apparatus (Bio-Rad Laboratories). Vacuum was subsequently applied to filter. Blotted membrane was washed with 2X SSC buffer and air-dried. The membrane was then blocked with 5% non-fat milk and incubated with anti 5hmC antibody (Active motif). Binding of an HRP-conjugated secondary antibody was visualized by chemiluminescence (Amersham ECL Western Blotting detection reagents). To control equal spotting of total DNA onto the membrane, the same blotted filter was then stained with 0.02% methylene blue in 0.3 M sodium acetate (pH 5.2). A total of 3.5 μg of DNA was used for samples deriving from untransfected HEK293T cells, 1.75 μg of DNA from TALE-TET1 FL samples and 350 ng of DNA from TALE-TET1 CD samples. Densitometric analysis was performed by Quantity One Software (Bio-Rad Laboratories) according to manufacturer's instructions.

### ELISA-based quantification of 5hmC

Colorimetric quantification of 5hmC was performed by using the Quest 5-hmC™ DNA ELISA Kit (Zymo research) according to manufacturer's instructions.

### RNA extraction and quantitative RT-PCR

Total RNA was isolated from cells by using RNeasy mini kit (Qiagen) following the manufacturer's instructions. RNase-free DNase (Qiagen) treatment was performed to eliminate contaminating DNA. Total RNA was subjected to reverse transcription using SuperScript VILO cDNA Synthesis Kit (Life Technologies). Transcriptional analysis was performed by quantitative RT-PCR (qRT-PCR) using iCycler IQ detection system (Bio-Rad). For quantitative PCR reactions, Taqman Gene Expression Assays (PARP-1 Hs00242302_m1; PARP-2 Hs00193931_m1; PARP-3 Hs00193946_m1; PARG Hs00608254_m1; GUSB Hs99999908_m1) and EXPRESS qPCR Supermix Universal (Life Technologies) were used. Measurement of gene expression was performed using the comparative cycle threshold method.

### Co-immunoprecipitation

Nuclei isolated from HEK293T cells were lysed in Co-IP buffer (50 mM Tris-HCl pH 7.4, 250 mM NaCl, 0.5% NP-40 and 2% glycerol) supplemented with protease inhibitors (Complete EDTA-free, Roche Applied Science). NaCl and glycerol concentrations were adjusted to 150 mM and 1%, respectively. Lysates were then pre-cleared with Protein G-agarose beads or Protein A-agarose beads (Millipore) on a rotating shaker at 4°C for 2.5 hrs. Pre-cleared lysates were incubated with specific antibodies or relative normal control IgGs (Santa Cruz Biotechnology) on a rotating shaker overnight at 4°C. Agarose beads, previously saturated with BSA (1 μg/μl) overnight, were added to the lysate/antibody solutions and incubated for 3 hrs on a rotating shaker at 4°C. Subsequently, beads were washed 10 times in Co-IP wash buffer (50 mM Tris-HCl pH 7.4, 150 mM NaCl, 0.5% NP-40 and 1% glycerol), proteins were eluted boiling in 2X Laemmli buffer and then analysed by Western blotting.

### GST pull-down

5 pmol of recombinant GST-TET1 or recombinant GST-tag (SignalChem) were incubated with 25 μl of PBS-washed Glutathione Sepharose 4B (GE Healthcare) for 1 hr in rotation at 4°C. After washing with equilibration buffer (50 mM Tris-HCl pH 8.0, 5 mM MgCl_2_), 5 pmol of unmodified recombinant PARP-1 (Enzo Life Sciences) or automodified recombinant PARP-1 were added to equilibration buffer and incubated with GST-TET1 or GST for 2 hrs in rotation at 4°C. Samples were washed 10 times with wash buffer (50 mM Tris-HCl pH 8.0, 5 mM MgCl_2_, 150 mM NaCl, 0.1% NP-40, 1% glycerol) and elution was obtained boiling with one volume of 2X Laemmli sample buffer. PARP-1 automodification was obtained incubating 5 pmol of recombinant PARP-1 in PARP activity buffer (50 mM Tris-HCl pH 8.0, 5 mM MgCl_2_, 0.2 mM DTT, 3 μM NAD^+^) in presence of 200 ng of DNAse I activated DNA (Enzo Life Sciences) for 45 min at 25°C. GST pull-down was also performed in presence of 300 μg of nuclear proteins with an incubation of 4 hrs in rotation at 4°C.

### Synthesis and purification of PARs

Purification of PARs was performed as previously described [62]. Briefly, 1.5 units of human recombinant PARP-1 (Enzo Life Sciences) were incubated for 2 hrs at 30°C in poly(ADP-ribosyl)ation buffer (100 mM Tris-HCl pH 8.0, 10 mM MgCl_2_, 20 mM DTT, 200 μM NAD^+^, 10% ethanol, 10% glycerol) with 500 ng of nicked DNA (Enzo Life Sciences). Reaction was stopped with 3 M sodium acetate (pH 5.2) and 0.7 volume of isopropanol and kept overnight at −20°C. Samples were then centrifuged for 30 min at 16000 x g and washed with 70% ethanol. The pellet was resuspended in 10 mM Tris-HCl (pH 8.0), 1 mM EDTA and 200 μg of proteinase K (Sigma-Aldrich) and incubated overnight at 37°C. Then, 1 volume of 1 M KOH/100 mM EDTA was added and the samples were incubated for 2 hrs at 37°C, centrifuged and PARs were recovered by chloroform/isoamyl alcohol/ethanol precipitation. Pellets were resuspended in RNase DNase-free water (Millipore).

### PAR blot assay

Recombinant proteins in equal molar amounts were dotted onto nitrocellulose membranes (Hybond ECL Amersham Pharmacia Biotech) in an assembled Bio-Dot apparatus (Bio-Rad). The blots were treated as described previously [62]. Briefly, blots were incubated in TBS-T (TBS-0.05% Tween 20) containing PARs with or without dsDNA (double-stranded DNA) of salmon sperm as competitor, at a PAR:dsDNA ratio of 1:25 (w/w). After incubation for 3 hrs at 21°C, membranes were extensively washed with TBS-T and subjected to immunoblotting using mouse monoclonal anti PAR antibody (10 HA, Trevigen) and goat anti-mouse horseradish peroxidase conjugated antibody (Santa Cruz Biotechnology). Recombinant H2B (histone 2B, Sigma–Aldrich) was used as positive control while GST as the negative one.

### *In vitro* TET enzymatic activity analysis

TET enzymatic activity was measured by using the ELISA-based Epigenase 5mC Hydroxylase TET Activity/Inhibition Assay Kit (Epigentek) according to manufacturer's instructions. Incubation time of TET1 recombinant enzyme/nuclear lysates was extended to two hrs.

### *In vitro* PARylation assay

7 pmol of GST-TET1 (SignalChem) or recombinant GST-tag were incubated with 25 μl of PBS-washed Glutathione Sepharose 4B (GE Healthcare) for 1 hr in rotation at 4°C. After washing with equilibration buffer (50 mM Tris-HCl pH 8.0, 5 mM MgCl_2_), 3.5 pmol of recombinant PARP-1 (Enzo Life Sciences) were added to PARP activity buffer (50 mM Tris-HCl pH 8.0, 5 mM MgCl_2_, 0.2 mM DTT, 3 μM NAD^+^) in presence of DNAse I activated DNA (Enzo Life Sciences) and incubated with GST-TET1 or GST at 25°C for different time. Reaction was stopped adding one volume of 2X Laemmli sample buffer. For *in vitro* TET activity of covalently PARylated GST-TET1, elution was performed in 50 mM Tris-HCl pH 8.0 and 20 mM reduced glutathione after extensive incubations with detergent containing buffer (50 mM Tris-HCl pH 8.0, 5 mM MgCl_2_, 200 mM NaCl, 0.1% NP-40, 1% glycerol) to wash out PARP-1 protein. Experiments were also performed without DNAse I activated DNA incubating 1 pmol of GST-TET1 or GST in presence of 0.4 pmol of PARP-1 for different time or incubating 9, 1.8, 2.7, 3.6 pmol of GST-TET1 or GST in presence of 9 pmol of PARP-1 for 15 min at 25°C.

### Statistics

Values are expressed as mean ± SEM. Paired Student *t*-test or ANOVA with post hoc Tukey's test were used to compare results between different groups. Significance was accepted at the level of *P* < 0.05.

## SUPPLEMENTARY MATERIAL FIGURES


