# Supplementary material for: 5mC-hydroxylase activity is influenced by the PARylation of TET1 enzyme
Source: Oncotarget. 2015 Jun 15;6(27):24333–47. doi: 10.18632/oncotarget.4476 (PMC4695189; doi:10.18632/oncotarget.4476)
Supplement: Supplementary file 1 [file oncotarget-06-24333-s001.pdf]

5mC-hydroxylase activity is influenced by the PARylation of TET1 enzyme

Supplementary Material

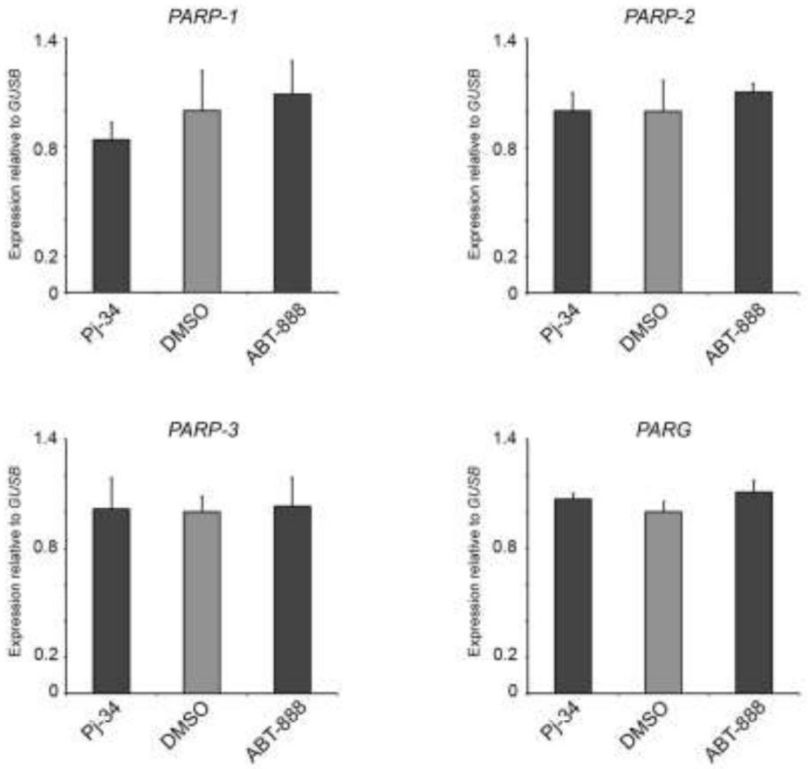

Figure S1: qRT-PCR analysis of *PARP-1*, *PARP-2*, *PARP-3* and *PARG* genes in HEK293T cells treated for 72 hrs with the PARP inhibitors Pj-34 and ABT-888. The results are shown as means ± S.E.M. (n=3). Statistical analysis was performed by One-way ANOVA test.

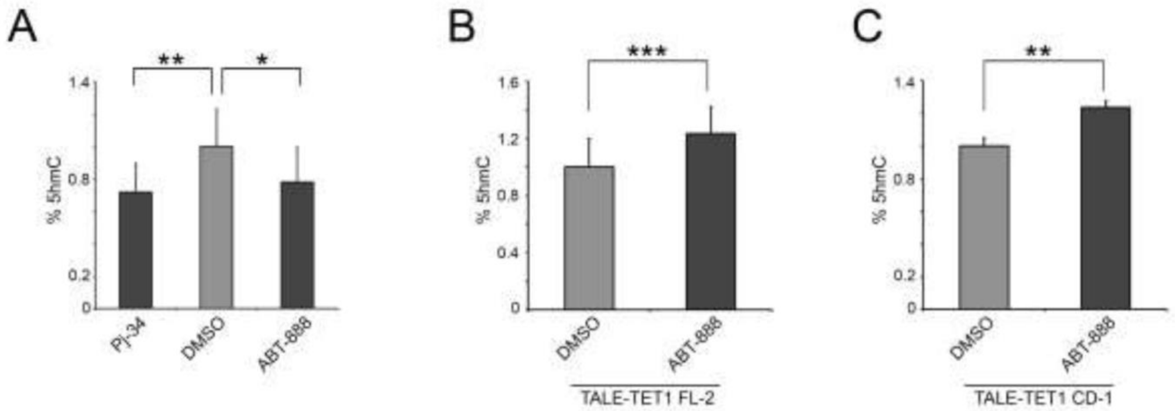

Figure S2: ELISA-based quantification of 5hmC performed on DNA obtained from (A) HEK293T cells treated for 72 hrs with the PARP inhibitors Pj-34 and ABT-888; (B) HEK293T cells overexpressing TALE-TET1 FL-2 and treated with/without ABT-888; (C) HEK293T cells overexpressing TALE-TET1 CD-1 and treated with/without ABT-888. The results are shown as means ± S.E.M. (n=3). Statistical analysis was performed by One-way ANOVA test (A) and paired Student's t-test (B and C) (*P<0.05; **P<0.01; **P<0.001).

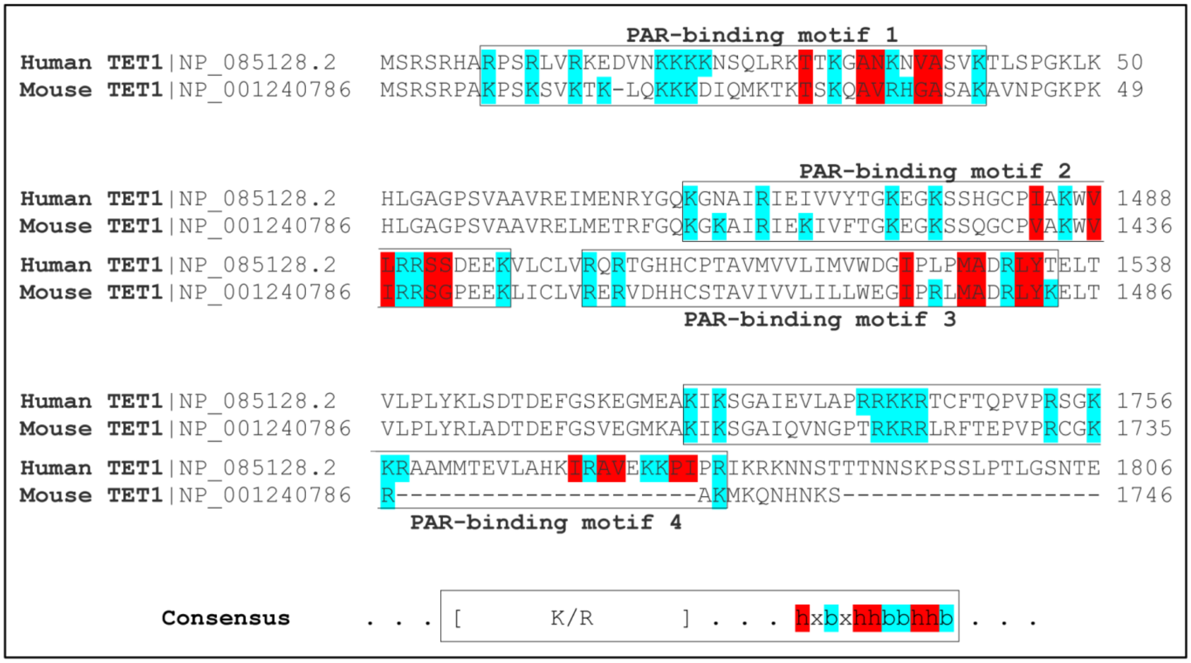

Figure S3: Alignment of the corresponding sequences of human and mouse TET1 proteins showing the putative PAR binding motifs. Aminoacid sequences were compared using CLUSTALW. PAR-binding-motifs are surrounded by black boxes. PAR-binding motif 1, 2 and 3 are conserved in both TET1 proteins. The PAR-binding motif 4 is specific for the human TET1 protein and present in TET1 catalytic domain. The PAR-binding motif consensus is reported and essential aminoacids are highlighted (blue: basic, red: hydrophobic).

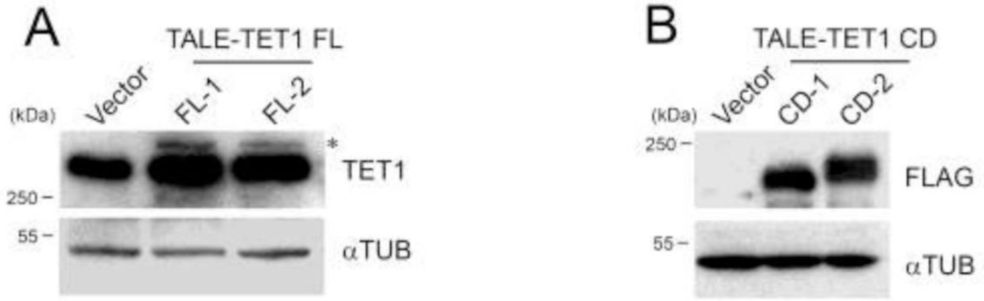

Figure S4: Western blot analysis demonstrating the overexpression in HEK293T cells of (A) TALE-TET1 FL-1 and FL-2 (asterisk) constructs and (B) TALE-TET1 CD-1 and CD-2 constructs. α-TUBULIN was used as loading control.

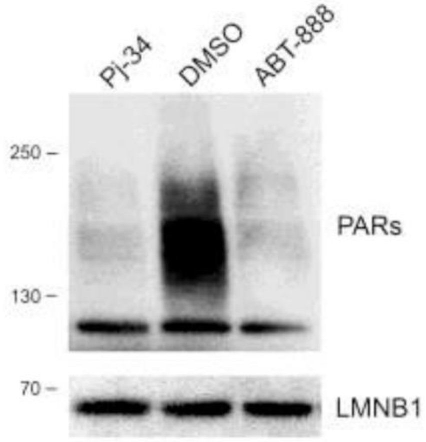

Figure S5: Western blot analysis performed on HEK293T lysates corresponding to Figure 5A and showing the depletion of PARs after treatment with the PARP inhibitors Pj-34 and ABT-888. LAMIN B1 (LMNB1) protein was used as loading control.
